# Rapid Detection of *Mycobacterium tuberculosis* by Recombinase Polymerase Amplification

**DOI:** 10.1371/journal.pone.0103091

**Published:** 2014-08-13

**Authors:** David S. Boyle, Ruth McNerney, Hwee Teng Low, Brandon Troy Leader, Ailyn C. Pérez-Osorio, Jessica C. Meyer, Denise M. O'Sullivan, David G. Brooks, Olaf Piepenburg, Matthew S. Forrest

**Affiliations:** 1 Program for Appropriate Technology in Health, Seattle, WA, United States of America; 2 Faculty of Infectious and Tropical Diseases, London School of Hygiene & Tropical Medicine, London, United Kingdom; 3 Washington State Department of Health, Public Health Laboratories, Shoreline, WA, United States of America; 4 TwistDx Limited, Cambridge, United Kingdom; San Francisco General Hospital, University of California San Francisco, United States of America

## Abstract

Improved access to effective tests for diagnosing tuberculosis (TB) has been designated a public health priority by the World Health Organisation. In high burden TB countries nucleic acid based TB tests have been restricted to centralised laboratories and specialised research settings. Requirements such as a constant electrical supply, air conditioning and skilled, computer literate operators prevent implementation of such tests in many settings. Isothermal DNA amplification technologies permit the use of simpler, less energy intensive detection platforms more suited to low resource settings that allow the accurate diagnosis of a disease within a short timeframe. Recombinase Polymerase Amplification (RPA) is a rapid, low temperature isothermal DNA amplification reaction. We report here RPA-based detection of *Mycobacterium tuberculosis* complex (MTC) DNA in <20 minutes at 39°C. Assays for two *MTC* specific targets were investigated, IS*6110* and IS*1081*. When testing purified *MTC* genomic DNA, limits of detection of 6.25 fg (IS*6110*) and 20 fg (IS1081)were consistently achieved. When testing a convenience sample of pulmonary specimens from suspected TB patients, RPA demonstrated superior accuracy to indirect fluorescence microscopy. Compared to culture, sensitivities for the IS*1081* RPA and microscopy were 91.4% (95%CI: 85, 97.9) and 86.1% (95%CI: 78.1, 94.1) respectively (n = 71). Specificities were 100% and 88.6% (95% CI: 80.8, 96.1) respectively. For the IS*6110* RPA and microscopy sensitivities of 87.5% (95%CI: 81.7, 93.2) and 70.8% (95%CI: 62.9, 78.7) were obtained (n = 90). Specificities were 95.4 (95% CI: 92.3,98.1) and 88% (95% CI: 83.6, 92.4) respectively. The superior specificity of RPA for detecting tuberculosis was due to the reduced ability of fluorescence microscopy to distinguish *Mtb* complex from other acid fast bacteria. The rapid nature of the RPA assay and its low energy requirement compared to other amplification technologies suggest RPA-based TB assays could be of use for integration into a point-of-care test for use in resource constrained settings.

## Introduction

Tuberculosis (TB) is an infectious disease that is very often difficult to diagnose. The World Health Organisation (WHO) estimates that during 2012 the global case detection rate for TB was 66%, suggesting that of the estimated 8.6 million incident cases that year almost 3 million cases were not diagnosed and notified [1]. Pulmonary TB, the most infectious form of the disease, is diagnosed by detecting *Mycobacterium tuberculosis* complex (MTBC) bacilli in samples of sputum expectorated by the patient. Smear microscopy, which is the primary test used for the diagnosis of pulmonary TB endemic countries [1], is a laborious and relatively insensitive test with a case detection rate of only 56 to 68% [2]. While microscopy can diagnose late stage TB disease, it performs poorly on specimens acquired from cases with HIV co-infection due to the reduced numbers of TB cells produced in a sputum specimen. Failure to detect and treat pulmonary disease in a timely manner results in onward transmission and the continuation of the epidemic which claimed an estimated 1.2 million lives during 2012 [1]. The WHO recently noted that in 2012 approximately three million cases of active TB went undiagnosed by country programs [1] and there is a pressing need for improved diagnostic tools to supplant smear microscopy to facilitate rapid detection [3], [4].

To this aim, the Xpert MTB/RIF (Cepheid, USA) was endorsed by WHO in 2010 to detect pulmonary disease in settings with a high incidence of TB/HIV co-infection or with high rates of drug resistance [5]. This polymerase chain reaction (PCR) based test is fully integrated into a modular system and can detect both TB infection and indicate the presence of resistance to one of the key anti-tuberculosis drugs, rifampicin. The key attribute of the GeneXpert system is its ease of use, as the majority of key processes are integrated and automated from DNA extraction to interpretation of a test result [6]. However, the GeneXpert requires consistent electricity and laboratory temperatures maintained under 30°C, which poses logistical challenges [7]. Furthermore, the cost of cartridges, instrumentation and maintenance threaten its sustainability in the longer term in high TB burden countries that currently rely on global donor assistance to provide this technology. Therefore, to increase access to timely and accurate diagnosis for more TB suspected patients, rapid, high performance TB diagnostic tests that can meet the logistical challenges of unsupervised use in limited infrastructure settings are required and at a price that is more comparable to smear microscopy [3], [8]–[10]. Other technologies have been developed for TB diagnosis in microscopy centers in high burden countries and are currently undergoing evaluation. These include semi-modular systems (Epistem Genedrive and Molbio EASYNAT) or methods with manual specimen processing and test determination (Eiken LoopAMP TB and Ustar Biotechnologies Easy NAT) [9], [11]–[15].

**Figure 1:**
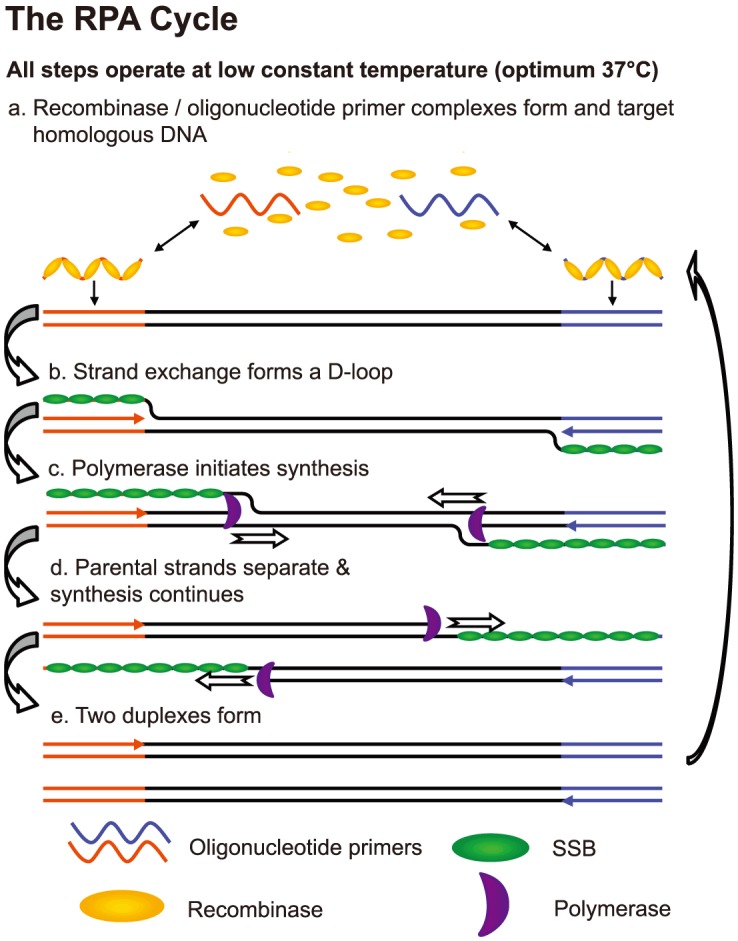
DNA amplification by Recombinase Polymerase Amplification. The three core proteins, recombinase, single-strand DNA binding protein (SSB) and strand-displacing polymerase enable PCR-like DNA amplification without the need for thermal cycling or an initial chemical or thermal melting step. This diagram was created by TwistDx Ltd (http://www.twistdx.co.uk/our_technology/) and is licensed under a Creative Commons Attribution 3.0 United States License.

Recently, isothermal amplification assays utilizing loop mediated amplification (LAMP), helicase dependant amplification (HDA) and cross priming amplification (CPA) have been described for the diagnosis of pulmonary TB [12], [16], [17]. Unlike PCR, isothermal assays do not require precisely controlled thermal cycling and instead use only a uniform incubation temperature, typically 55–65°C, to permit DNA amplification and therefore may offer greater utility with more simplistic reactor designs or heat sources [12], [18]–[20]. Recombinase Polymerase Amplification (RPA) has emerged as a novel, isothermal technology for use in molecular diagnosis of infectious disease [21]. Unlike many other isothermal technologies, RPA does not require elevated or precise temperatures, and may proceed at temperatures between 25°C and 42°C [21]. RPA replaces the thermal cycling needed for PCR with three core enzymes. The titular enzyme, a recombinase, binds to oligonucleotide primers to form recombination filaments that can recombine with homologous DNA (Figure 1). The second enzyme, a single-stranded DNA binding protein, binds to the strand of DNA displaced by the primer, stabilising the D-loop that has formed and preventing the dissociation of the primer. The third core enzyme is a strand-displacing DNA polymerase that copies the DNA template by adding bases onto the 3′ end of the primer, forcing open the DNA double helix as it progresses. As with PCR, the use of closely spaced opposing primers allows the exponential amplification of a defined region of DNA. RPA reactions typically run to completion in 5–15 minutes, depending on amplicon size and the template copy number [21]. This time to test result is significantly more rapid than the other isothermal MTBC assays which note incubation times in the range of 45–60 minutes [12], [13], [16].

The highly specific detection of RPA products is achieved via the use of custom designed oligonucleotide probes which recognize a complementary region within either strand of the RPA amplicon. The probe DNA is also inserted via a recombinase mediated event. RPA probes are constructed with an abasic nucleotide analogue within the probe sequence. This abasic site is recognised and cleaved by several types of endo- or exonucleases, but only when the probe has bound to its complementary sequence. Different nucleases are specifically added to the core RPA reaction mixture for use in a variety of detection formats, including fluorescence detection in real time or endpoint detection via a lateral flow strip [21], [22]. Reverse transcriptase, can be also included in RPA reactions to facilitate RPA from RNA targets such as Middle East Respiratory Syndrome Coronavirus or Rift Valley Fever [23], [24]. Recently RPA was been demonstrated to be highly sensitive for the detection of HIV proviral DNA [22] and in this work we have investigated the use of RPA to detect *Mycobacterium tuberculosis* DNA from patients presenting with suspected pulmonary TB.

## Materials and Methods

### RPA assay design

Oligonucleotide primers and probes used to develop the RPA assays were purchased from Eurogentec Ltd (Southampton, UK) and Biosearch Technologies (Novato, CA) respectively. Preliminary screening of primer and probe combinations used the Twist Amp Exo kits according to the manufacturer's instructions (TwistDx Ltd., UK) in final reaction volumes of 50 µL. The RPA reactions were incubated at 39°C using a combined heating and fluorescence detection device (Twista, TwistDx, UK) [22]. Samples were mixed prior to amplification and also at six minutes during incubation. The output of the RPA reaction was monitored in real time using Twista Studio Software (TwistDx, UK) with fluorescence measurements taken every 20 seconds for a total of 20 minutes.

Once optimal primer and probe sequences were identified, lyophilized pellets of the TwistAmp Exo reactions were prepared that also contained the primers and probe. For 50 µL reactions, 4 µL of 280 mM MgOAc, 37.5 µL TwistAmp Primer-In-Pellet Resuspension Buffer (PIRB, TwistDx Ltd) and 8.5 µL of sample/water were used to rehydrate the pellets. Double amounts of reagent were used for 100 µL reactions. Sample incubation conditions remained the same as used for primer screening.

### DNA Samples

Cultures of *M. tuberculosis* (H37Rv) and *M. bovis* BCG-*Bulgaria* (BB-NCIPD Ltd., *Bulgaria*) were maintained on Lowenstein Jensen slants supplemented with glycerol (Media for Mycobacteria, UK) and Middlebrook 7H9 media supplemented with 10% ADC (BD BBL, USA). DNA was extracted using the standardised RFLP protocol and quantified using the Qubit platform (Invitrogen, Life Technologies Ltd. UK) [25]. Samples of purified DNA from non tuberculous mycobacteria (NTM) were supplied by the Mycobacteriology Unit (Institute of Tropical Medicine, Antwerp, Belgium [Table 1]). DNA concentrations were quantified using the Qubit platform (Invitrogen, Life Technologies Ltd. UK) and 500 pg (∼114,000 genomes) samples in a volume of 1 µL were added to the RPA reactions. Using the molecular weight per genome as determined from the whole genome sequence of the BCG Tokyo 172 strain (Accession #: 224771496) we calculate that 5 fg of BCG DNA correlates to an estimated genome equivalent (GE) of one cell [26]. A second panel of fully characterized NTM strains and other bacteria including common respiratory pathogens were accessed from the reference collection of the Washington State Public Health Laboratory (Table 1). Crude genomic DNA extracts were prepared as described previously [27].

**Table 1:** Strains used for specificity testing.

*M. absessus* ATCC 19977	*M. mageritense* 981294	*Arcanobacterium hemolyticum*
*M. aurum* ATCC 23366*	*M. malmoense* ATCC 29571	*Bordetella cepacia* ATCC 25410
*M. avium* 961091*	*M. malmoense* 40445*	*B. pertussis*
*M. avium* ATCC 25291	*M. marina* ATCC 927	*B. bronchiseptica* ATCC 19395
M. *brisbanense*	*M. marinum*1717	*Corynebacterium striatum*
*M. catarrhalis*	*M. nebraskense*	*Escherichia coli* ATCC 25922
*M. chelonae*	*M. neoaureum* 40853	*Gordonia bronchialis*
*M. chelonae* 41426	*M. nonchromogenicum* 970454*	*Haemophilus influenzae*
*M. chitae* ATCC 19628*	*M. nonchromogenicum* ATCC 19530	*Legionella pneumophilia* ATCC 33152
*M. diernhoferi* ATCC 19340*	*M. parafinicum* 3098*	*Neisseria meningitidis* ATCC 13077
*M. fortuitum* ATCC 6841	*M. peregrinum*	*Pseudomonas aeruginosa*
*M. gordonae* ATCC 14470	*M. peregrinum* 10086*	*Rhodococcus equi*
*M. gordonae*10284	*M. poriferae* ATCC 35087	*Staphylococcus aureus* ATCC 29213
*M. heidelbergense* 981295*	*M. scrofulaceum* ATCC 19073*	*Streptococcus agalactiae* ATCC 27591
*M. immunogen* 981291*	*M. shimodei* 27962*	*S. anginosus* ATCC 33397
*M. intracellulare* 32665*	*M. simiae* ATCC 25273	*S. pneumoniae* ATCC49619
*M. intracellulare* ATCC 13950	*M. smegmatis* mc2155*	*S. pyogenes* ATCC 12370
*M. intracellul*are ATCC 35761	*M. szulgai* ATCC 35799	*S. salivarus*
*M. kansasii*	*M. szulgai* 4981*	*Yersinia pseudotuberculosis* ATCC 23207
*M. kansasii* ATCC 12478	*M. ulcerans* 5147	
*M. kansasii* MYC 4296	*M. xenopi*	
*M. lentifalvum* 960190*	*M. xenopi* 9741*	
*M. liflandi* 40413*		

Non-tuberculous mycobacterial (NTM) strains and other bacterial species used in the determination of the specificity of the IS*6110* and IS*1081* RPA assays. All tested negative by both RPA assays. Isolates marked * were supplied by the Mycobacteriology Unit, Institute of Tropical Medicine, Antwerp, Belgium. All other isolates were from the Washington State Public Health Laboratory strain collection.

### Clinical samples

An evaluation of the RPA assay was performed using a panel of sputum and respiratory specimens derived from suspected cases of pulmonary TB obtained from the Washington State Public Health Laboratories. Samples tested by RPA were remains of specimens collected for routine diagnosis and were not representative of the population attending the clinic having been selected to provide a panel with an unusually high proportion of positive samples. Study investigators were blinded to the status of the samples. The panel comprised 121 specimens including induced and expectorated sputum (n = 119) and respiratory washes (bronchial and tracheal, n = 2) collected from a total of 101 TB suspect cases (no more than two specimens per individual were tested). Specimens were processed via digestion and decontamination using the N-acetyl L-cysteine NaOH-Na citrate method [28]. For each specimen, cells were pelleted by centrifugation at 6000× *g* and the supernatant was discarded. Each pellet was then resuspended in 1.5 mL of sterile phosphate-buffered saline (PBS, pH 6.8). Smears were prepared from the decontaminated specimens to determine the presence acid fast bacteria using Auramine O and fluorescence microscopy. A specimen was considered smear positive if greater than 1 AFB was present per 10 observed microscopic fields. The smear positive specimens were scored from +/− (scanty), 1+ to 4+ based on the number of observed AFB positive cells present [28].

Culture was performed using MGIT modified Middlebrook 7H-9 liquid medium in an automated BACTEC 460TB System (Becton, Dickinson and Company, New Jersey, USA) with incubation at 37°C for up to 1 month. A 0.5 mL inoculum of processed specimen was used per mycobacterial culture. All positive cultures were then screened for MTBC using the Mycobacterium Tuberculosis Complex Culture Identification Test (Gen-Probe, San Diego, CA) according to the manufacturer's instructions. The MTBC negative culture positive isolates were speciated by additional biochemical testing. A 250 µL aliquot of each processed specimen was used for DNA extraction. A previously described extraction process using glass beads and heat lysis was used, the lysed samples were then stored at −20°C until use [27]. The specimens screened by RPA TB assays were prepared as described earlier. Each RPA reaction contained 5 µL of specimen extract added with rehydration buffer, MgOAc and molecular grade water to create a final reaction volume of 50 µL. Scoring of positive RPA reactions incubated in the Twista RPA reactor was indicated by an increase in FAM fluorescence by greater than 300 mV from six to twenty minutes incubation.

The clinical materials used in this study were discarded remains of specimen collected for routine diagnostic examination, all data was blinded with no links to enable identification of patients. As such, the study was exempt from ethical approval in accordance with US Code of Federal Regulations 45 CFR 46.101. Work in the UK was approved by the Research Ethics Committee of the London School of Hygiene & Tropical Medicine and was undertaken in compliance with the Human Tissue Act (2004).

## Results

### Design of RPA assays for IS6110 and IS1081

An RPA assay was designed to target the insertion sequence, IS*6110* [29], [30], a region that has been shown to have high sensitivity for diagnosing tuberculosis using PCR [31], [32]. IS*6110* is often present in *M. tuberculosis* in multiple copies of up to 25 per genome [33] but conversely MTBC isolates with no IS*6110* target have also been described [34], [35]. *Mycobacterium bovis* bacillus Calmette-Guérin (BCG) strains have a low number of IS*6110* copies, the Bulgarian BCG strain used in this study having two copies [36]. To ensure coverage of all MTBC species, an second RPA assay for the insertion sequence IS*1081* was also developed [37]. Unlike IS*6110*, IS*1081* is present in all MTBC species and at a more stable copy number of 5–7 repeats per genome [38], the Bulgarian BCG strain used in this study having six copies. The primers and probes for both the IS*6110* and IS*1081* assays were selected based upon sequences described for the MTB H37Rv whole genome (NCBI Reference Sequence: NC_000962.3 [39]) and M. bovis BCG [37]. All initial designs were then screened using BLASTN and the NCBI nucleotide database to ensure the target sequences for the primers and probes were exclusive to MTBC species only [40]. The only difference between RPA primers and PCR primers is one of length, with 30–38 base oligonucleotides being optimal for the formation of efficient recombinase filaments. TwistAmp exo probes are oligonucleotides that are typically 46–52 bases long, with a tetra-hydrofuran (THF, sometimes referred to as a dSpacer) creating an abasic site in the oligonucleotide probe sequence [21]. The THF is positioned ≥30 bases from the 5′ end and ≥15 bases from the 3′ end. A fluorophore and a quencher are positioned on either side of the THF moiety, typically 2–4 bases apart. The only commercially available labelled phosphoramidite bases are dT and so the location of T nucleosides dictates the precise location of probes. The 3′ ends of each probe are blocked with a C3-spacer to prevent DNA polymerase extension from undigested probes. However, after exonuclease III digestion at the abasic site within the Exo probe, the newly generated 3′ end of the larger portion of the cleaved probe has a hydroxyl group from which DNA polymerase mediated strand extension can then occur.

Once probes had been selected, ten 35 mer primers were chosen at 5 base intervals (30 base overlap) upstream and downstream of the target regions for screening with 50 copies of TB DNA. Screening was performed with TwistAmp Exo reactions. After the initial screens, the primers that gave the best amplification of target DNA were then redesigned as a series of staggered primers with single base intervals deviating from the original primer sequence. These sets were then screened in in an effort to identify if any produced a faster time to result from 50 copies of template DNA. A third and final round of primer screening was performed with different length (30–38 bases, varying at the 3′ end) versions of the best primers. Figure S1 shows how this screening can improve performance of an assay. The oligonucleotides selected to amplify IS*6110* and IS*1081* are listed in Table 2.

**Table 2:** Oligonucleotide primers and probes.

Oligonucleotide	Sequence (5′ → 3′)
IS6110c_Forward	GATCCTGCGAGCGTAGGCGTCGGTGACAAAGGCCACGTAG
IS6110c_Reverse	CTGATCCGGCCACAGCCCGTCCCGCCGATCTCGTCCAGC
IS6110c_Probe	CGAACCCTGCCCAGGTCGACACATAGGTGAGGTC(F)(H)C(Q)ACCCACAGCCGGTTA-Spacer C3
IS1081a_Forward	CAGTAGTGGGCGGTCATCGCGTGATCCTTCGAAACGACC
IS1081a_Reverse	CTCGCCTGTGCGAGTTGGTCAGCCAGAAGCTG
IS1081a_Probe	CGATAAGATGAGAAGAGGTCATTGCGTCATT(F)(H)C(Q)TCGATTGACTTTTGCT-Spacer C3

The oligonucleotides chosen for amplification and detection of IS*6110* and IS*1081* are shown in Table 2. F = dT-FAM, H = tetra hydrofuran and Q = dT-Black Hole Quencher 1.

### Analytical sensitivity of RPA

**Figure 2:**
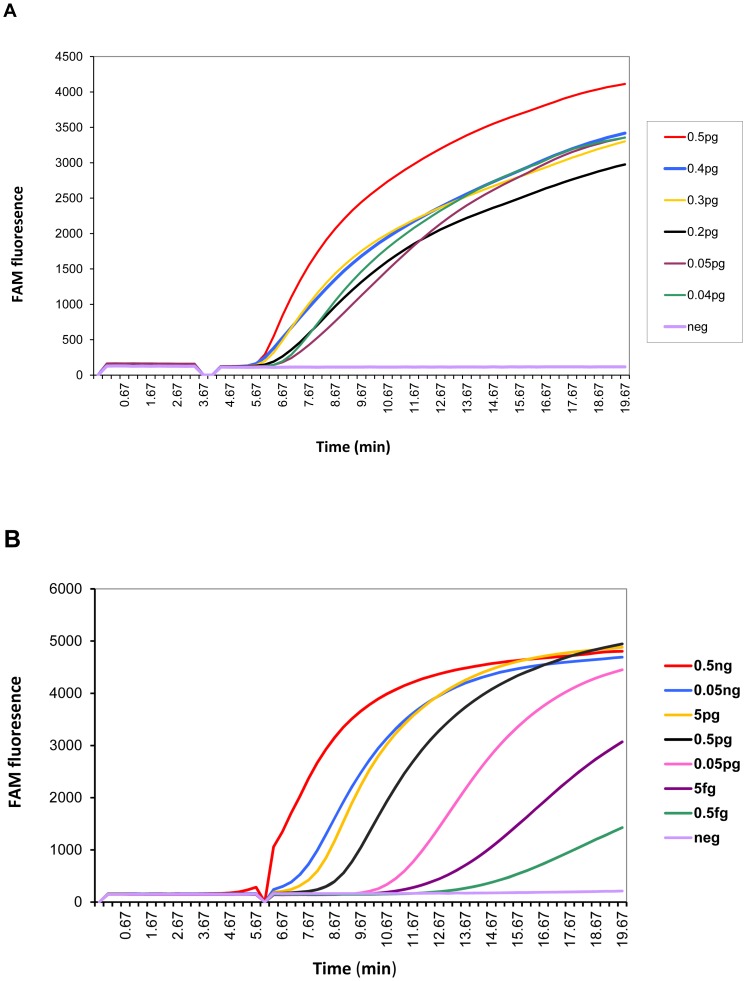
Real-time RPA amplification of IS*1081* and IS*6110*. Figure **2a** shows the real time detection of IS*1081* amplification from a dilution series of quantitated *M. bovis* BCG DNA with a sensitivity as low as 0.04 pg of DNA per reaction. Figure **2b** shows the real time detection of IS*6110* amplification from a dilution series of quantitated *M. bovis* BCG DNA BCG DNA with a sensitivity as low as 5.0 fg of DNA per reaction.

The sensitivity of the RPA reactions were assessed by testing serial dilutions of genomic DNA extracted from *M. bovi*s BCG. Multiple tests were performed at each concentration of DNA, with water used as a negative control (Table 3). An example of the output is shown in Figures 2a and 2b. The time to onset of a rise in fluorescence increased as the amount of BCG genomic DNA in the sample decreased. When using IS*6110* primers, 6.25 fg per 100 µL reaction were required for reactions to be consistently positive, which is approximately equivalent to the DNA found in a single bacteria (n = 9). As shown in figure 2b, when testing low levels of DNA the time to onset of fluorescence was associated with the amount of DNA in the samples, with 11 min being required when testing 5 fg. When using IS*1081* primers consistent positive results were obtained for all samples containing at least 20 fg BCG genomic DNA (Table 3). The time to onset of signal at the lowest concentration of 20 fg ranged from 7 to 9 minutes. These results suggested that either RPA assay is highly sensitive for the rapid amplification and detection of MTBC DNA, and that a 15 minute data collection period is sufficient to detect positive samples. Sensitivity was also assessed by testing replicate serial dilutions of a culture of BCG. Results (not shown) indicated that both RPA assays were capable of detecting a single colony forming unit of bacteria.

**Table 3:** Limit of detection of IS*6110* RPA and IS*1081* RPA.

DNA amount	0 fg	0.5 fg	0.78 fg	1 fg	1.56 fg	3.13 fg	5 fg	6.25 fg	10 fg	12.5 fg	20 fg	25 fg	30 fg	40 fg	50 fg	100 fg	500 fg	1 pg	5 pg	10 pg	50 pg	100 pg	0.5 ng	1 ng
IS*6110* positive/tested	0/8	2/5	0/4	N/A	0/4	1/4	2/4	4/4	N/A	4/4	N/A	4/4	N/A	N/A	8/8	N/A	5/5	N/A	4/4	3/3	4/4	N/A	4/4	N/A
IS*1081* positive/tested	0/8	N/A	N/A	0/3	N/A	N/A	0/2	N/A	5/8	N/A	5/5	N/A	5/5	5/5	5/5	3/3	3/3	3/3	N/A	3/3	N/A	3/3	N/A	3/3

Results shown are for purified DNA extracted from *M. bovis* BCG. DNA concentrations were measured prior to the preparation of serial dilutions for testing by RPA. The numbers of positive test results and the total number of tests run at each concentration of DNA are recorded.

### Specificity of RPA

To assess the specificity of the IS*6110* and IS*1081* RPA reactions for the identification of MTBC, samples of DNA from a panel of 23 non tuberculous mycobacteria (NTMs) were tested (Table 1). Positive controls using 500 pg DNA from either *M. bovis* BCG or *M. tuberculosis* H37Rv were used for each batch. No signals were obtained from the bacterial DNAs other than the MTBC strains, indicating high specificity for the primers used. In a second round of screening to challenge the RPA TB assay's specificity, genomic DNA from a further 25 NTMs and 19 non-mycobacterial species was used (Table 1). The crude DNA extracts derived from cultured bacteria indicated no cross reactivity with RPA assay for any bacterial species further confirming the high specificity of the IS*6110* and IS*1081* assays across a broad range of NTMs, or more divergent bacterial species.

### Assessment of RPA performance using clinical specimens

#### *IS6110* RPA assay

The specimen panel used to assess RPA assay performance was biased in its construction to reflect approximately 50% MTBC positive and 50% MTBC negative samples respectively, based on liquid culture results with subsequent pathogen identification. Fifty-six specimens were culture positive and of these forty-five were confirmed as MTBC positive after confirmation via Accuprobe testing (Table S1). The remaining eleven cultures were further identified as NTMs. Thirty-one specimens were culture negative and three cultures were contaminated (See Table S1). For these three, new specimens were acquired, and all three were later confirmed as MTBC positive by culture and Accuprobe. Smear microscopy results for this panel observed AFB ranging from 4+ to scanty for thirty-nine specimens while fifty-one were smear negative [41]. From this panel forty-four samples were found positive by RPA (Table 4, Table S1) and the sensitivity and specificity of the IS*6110* RPA assay when compared to confirmed culture were 87.5% (95% CI: 81.7,93.2) and 95.4 (95% CI: 92.3,98.1) respectively. The indirect smear microscopy was less accurate at 70.8% (95% CI: 62.91, 78.75) and 88% (95% CI: 83.6, 92.4). The *IS1081* RPA assay detected 100% of smear positive TB samples, and 8/14 (57.1%; 95%CI: 33.8, 80.4) of the smear negative culture confirmed TB specimens (Table 5, Table S1). Four NTMs were found smear positive but RPA negative.

**Table 4:** Tuberculosis detection by indirect smear microscopy vs RPA IS*6110*.

	Culture positive	Culture negative
Indirect smear microscopy positive	34	5
Indirect smear microscopy negative	14	37
RPA *IS6110* positive	42	2
RPA *IS6110* negative	6	40

Testing pulmonary specimens (n = 90) by indirect smear microscopy and RPA IS*6110* to detect tuberculosis, with comparison to liquid culture based test data. RPA *IS6110* was more sensitive than indirect smear microscopy (87.5% (95% CI: 81.7, 93.2) vs 70.8% (95% CI: 62.91, 78.75)) and also more specific (95.4 (95% CI: 92.3, 98.1) vs 88% (95% CI: 83.6, 92.4)).

**Table 5:** Tuberculosis detection by indirect smear microscopy vs RPA IS*1081*.

	Culture positive	Culture negative
Indirect smear microscopy positive	31	4
Indirect smear microscopy negative	5	31
RPA *IS1081* positive	32	0
RPA *IS1081* negative	4	35

Testing pulmonary specimens (n = 71) by indirect smear microscopy and RPA IS*1081* to detect tuberculosis, with comparison to liquid culture based test data. RPA *IS1081* was more sensitive than indirect smear microscopy (91.4% (95% CI: 85,98.9) vs 86.1% (95% CI: 78.1, 94.1)) and also more specific (100% vs 88.6% (95% CI: 80.8, 96.1)).

#### *IS1081* RPA assay

The panel used to assess the performance of the IS*1081* RPA assay also contained a disproportionately high number of MTBC positive specimens. A total of 71 specimens previously characterized by smear microscopy and culture were used. Due to limited access to clinical materials only forty of the specimens used in the IS*6110* assay evaluation were also used to evaluate the IS*1081* RPA assay. The remaining 31 isolates were only screened with the IS*1081* RPA assay. The panel used contained 47 culture positive samples of which 35 were confirmed as MTBC via Accuprobe, and 12 as NTMs (Table 4, Table S1). The remaining 24 samples were culture negative. According to smear microscopy data, this panel contained 36 smear positive specimens ranging from 4+ to scanty (+/−). From this panel 32 samples were found positive by RPA (Table 4, Table S1) and the sensitivity and specificity of the IS*1081* RPA assay when compared to confirmed culture were 91.4% (95% CI: 85,98.9) and 100% respectively. The indirect smear microscopy was less accurate at 86.1% (95% CI: 78.1, 94.1) and 88.6% (95% CI: 80.8, 96.1). RPA detection of smear positive TB samples was 96.8% and 2/5 (40%; 95%CI: 0,98.8) of the smear negative samples from culture confirmed TB patients were detected. Four NTMs were found smear positive but RPA negative.

## Discussion

In this study we have demonstrated the application of RPA to diagnosis TB from sputum. Two assays targeting separate repetitive elements were examined, both demonstrating the ability to detect MTBC DNA with a high degree of sensitivity and specificity in less than 20 minutes. Of the two assays the IS*6110* RPA consistently detected ∼6.5 fg of genomic DNA, and thus could detect samples estimated to contain the genome equivalent of a single bacterial cell. The IS*1081* assay required slightly higher amounts of DNA 20 fg, which was not expected as it does not reflect the increased number of copies of this insertion element in the genome (six IS*1081* vs two IS*6110*). We suspect the lower sensitivity observed is due to reduced primer and probe binding efficiency of the oligonucleotides screened in this study. We also cannot rule out the possibility of localized secondary or tertiary DNA structures affecting the recombinase mediated insertion of the oligonucleotides despite the presence of high levels of ssDNA binding protein in RPA reactions. Nonetheless both assays demonstrated the potential of RPA as a diagnostic tool to detect tuberculosis in samples containing low numbers of bacteria. Both assays were shown to be highly specific for MTBC (100%) when challenged with acid fast bacterial species that do not cause tuberculosis or 18 other common commensal or respiratory bacterial pathogens.

When the performance RPA was assessed with clinically derived specimens, we found that both assays had high sensitivity (100%) with isolates that were smear and culture positive. For samples that were smear negative but culture positive 8/14 (57.1%) and 2/5 (40%) were identified as positives via the IS*6110* and IS*1081* assays, respectively. It should be noted a crude DNA extraction procedure was used and improved sample handling and DNA extraction may improve detection from complex sample matrixes such as sputum [42]. Similarly whereas 0.5 ml inoculums were used for culture the volume taken for DNA extraction was only 0.25 ml, with just 5 µL being used for each RPA assay and there may be opportunity to improve sensitivity by testing larger sample volumes or by incorporating a sample concentration step. To fulfil the full potential of RPA for point-of-care diagnostics, an integrated platform to perform sample preparation and run the assay must be developed.

Further work is required to optimize specificity of RPA for TB diagnosis from clinical samples. In particular the number of negative samples included in this study were insufficient to assess the clinical specificity and the number of culture-positive, smear-negative samples should be increased to better determine the clinical sensitivity. Ten of ten NTM positive specimens were correctly assigned by the IS*1081* assay as being MTBC negative, demonstrated a specificity of 100%. However, whereas the IS*6110* assay correctly assigned two NTM specimens as negative two false positives were observed for smear negative specimens that were culture positive for NTM. The positive predictive value (PPV) and negative predictive value (NPV) are not presented as the contrived composition of the MTBC screening panels used in this study were selected to demonstrate proof of concept and do not reflect the normal distribution of specimens received for routine screening of pulmonary MTBC infection [43].

In this study we have provided an evidence base for RPA as a tool for the rapid and sensitive detection of DNA from MTBC. The comparatively low isothermal incubation temperature combined with rapid time to result allows RPA to be used with a battery powered device, making it amenable to health clinics in developing countries that do not have reliable electricity supply [44]. However, further studies are required to adapt sample extraction methodology for use at the point of care.

## Supporting Information

Figure S1 **Benefits of RPA primer optimisation.** This figure shows a side-by-side comparison of duplicates of the optimal primer pairs chosen during each round of screening for *IS6110* with either 50 (dotted lines) or 500 (solid lines) copies of template. The primers chosen at the end of the first round of screening (purple, ‘Round 1’), can detect both 50 and 500 copies of template. The optimal primers identified by shuffling these primers upstream and downstream in single base increments (red, ‘Shuffled’) give faster detection times and result in higher levels of fluorescence. The primers chosen from lengthening and shortening the 3′ end of these shuffled primers (green, ‘Length’) show further improvement to detection time and fluorescent signals. Note that in all instances, poor duplicates are often indicative that an assay is close to its limit of detection.
(TIF)

Table S1 **Culture, smear and RPA test results derived from clinical specimens.** The scores for bacteria counted in the smear +ve specimens are shown in parenthesis, e.g. +ve (2+). Smear scores that were scanty are shown as +/−. Acronyms and other points. MTBC – Mycobacterium tuberculosis complex; NT – Not Tested; NTM  = - Non Tuberculous Mycobacteria. MTBC. * Primary culture was contaminated; specimen was confirmed via a fresh specimen; # ***bronchoalveolar*** lavage (others not marked are sputum).
(DOCX)

## References

[1] World Health Organization (2013) Global Tuberculosis Report.

[2] World Health Organization (2009) Global Tuberculosis Report.

[3] McNerney R, Daley P (2011) Towards a point-of-care test for active tuberculosis: obstacles and opportunities. Nat Rev Microbiol 9: 204–213. 21326275 10.1038/nrmicro2521

[4] WHO (2010) The global plan to stop TB 2011–2015: transforming the fight towards elimination of tuberculosis.

[5] World Health Organisation (2010) Strategic and Technical Advisory Group for Tuberculosis Report of 10th Meeting. Geneva.

[6] Lawn SD, Nicol MP (2011) Xpert(R) MTB/RIF assay: development, evaluation and implementation of a new rapid molecular diagnostic for tuberculosis and rifampicin resistance. Future Microbiol 6: 1067–1082. 21958145 10.2217/fmb.11.84 PMC3252681

[7] Denkinger CM, Nicolau I, Ramsay A, Chedore P, Pai M (2013) Are peripheral microscopy centres ready for next generation molecular tuberculosis diagnostics? Eur Respir J 42: 544–547. 23904551 10.1183/09031936.00081113

[8] Abebe G, Paasch F, Apers L, Rigouts L, Colebunders R (2011) Tuberculosis drug resistance testing by molecular methods: opportunities and challenges in resource limited settings. J Microbiol Methods 84: 155–160. 21129417 10.1016/j.mimet.2010.11.014

[9] Niemz A, Boyle DS (2012) Nucleic acid testing for TB at the point-of-care in high burden countries. Exp Rev Mol Diagn 12: 687–701. 10.1586/erm.12.71 PMC3740172 23153237

[10] Pai NP, Pai M (2012) Point-of-care diagnostics for HIV and tuberculosis: landscape, pipeline, and unmet needs. Discov Med 13: 35–45. 22284782

[11] Castan P, de Pablo A, Fernandez-Romero N, Rubio JM, Cobb BD, et al (2014) Point-of-Care System for Detection of Mycobacterium tuberculosis and Rifampin Resistance in Sputum Samples. J Clin Microbiol 52: 502–507. 24478480 10.1128/JCM.02209-13 PMC3911309

[12] Fang R, Li X, Hu L, You Q, Li J, et al (2009) Cross-priming amplification for rapid detection of Mycobacterium tuberculosis in sputum specimens. J Clin Microbiol 47: 845–847. 19116359 10.1128/JCM.01528-08 PMC2650920

[13] Mitarai S, Okumura M, Toyota E, Yoshiyama T, Aono A, et al (2011) Evaluation of a simple loop-mediated isothermal amplification test kit for the diagnosis of tuberculosis. Int J Tuberc Lung Dis 15: 1211–1217. 21943848 10.5588/ijtld.10.0629

[14] Nikam C, Jagannath M, Narayanan MM, Ramanabhiraman V, Kazi M, et al (2013) Rapid diagnosis of Mycobacterium tuberculosis with Truenat MTB: a near-care approach. PLoS One 8: e51121. 23349670 10.1371/journal.pone.0051121 PMC3549918

[15] UNITAID (2012) Tuberculosis diagnostics technology & market landscape – semi-annual update.

[16] Ao W, Aldous S, Woodruff E, Hicke B, Rea L, et al (2012) Rapid detection of rpoB gene mutations conferring rifampin resistance in Mycobacterium tuberculosis. J Clin Microbiol 50: 2433–2440. 22518852 10.1128/JCM.00208-12 PMC3405577

[17] Boehme CC, Nabeta P, Henostroza G, Raqib R, Rahim Z, et al (2007) Operational feasibility of using loop-mediated isothermal amplification for diagnosis of pulmonary tuberculosis in microscopy centers of developing countries. J Clin Microbiol 45: 1936–1940. 17392443 10.1128/JCM.02352-06 PMC1933042

[18] LaBarre P, Hawkins KR, Gerlach J, Wilmoth J, Beddoe A, et al (2011) A simple, inexpensive device for nucleic acid amplification without electricity-toward instrument-free molecular diagnostics in low-resource settings. PLoS One 6: e19738. 21573065 10.1371/journal.pone.0019738 PMC3090398

[19] Mori Y, Nagamine K, Tomita N, Notomi T (2001) Detection of loop-mediated isothermal amplification reaction by turbidity derived from magnesium pyrophosphate formation. Biochem Biophys Res Commun 289: 150–154. 11708792 10.1006/bbrc.2001.5921

[20] Niemz A, Ferguson TM, Boyle DS (2011) Point-of-care nucleic acid testing for infectious diseases. Trends Biotechnol 29: 240–250. 21377748 10.1016/j.tibtech.2011.01.007 PMC3746968

[21] Piepenburg O, Williams CH, Stemple DL, Armes NA (2006) DNA detection using recombination proteins. PLoS Biol 4: e204. 16756388 10.1371/journal.pbio.0040204 PMC1475771

[22] Boyle DS, Lehman DA, Lillis L, Peterson D, Singhal M, et al (2013) Rapid detection of HIV-1 proviral DNA for early infant diagnosis using recombinase polymerase amplification. MBio 4. 10.1128/mBio.00135-13 PMC3622927 23549916

[23] Abd El Wahed A, El-Deeb A, El-Tholoth M, Abd El Wahed KH, Ahmed A, et al (2013) A portable reverse transcription recombinase polymerase amplification assay for rapid detection of foot-and-mouth disease virus. PLoS One 8: e71642. 23977101 10.1371/journal.pone.0071642 PMC3748043

[24] Abd El Wahed A, Patel P, Heidenreich D, Hufert FT, Weidmann M (2013) Reverse transcription recombinase polymerase amplification assay for the detection of middle East respiratory syndrome coronavirus. PLoS Curr 5. 10.1371/currents.outbreaks.62df1c7c75ffc96cd59034531e2e8364 PMC3871419 24459611

[25] van Embden JD, Cave MD, Crawford JT, Dale JW, Eisenach KD, et al (1993) Strain identification of Mycobacterium tuberculosis by DNA fingerprinting: recommendations for a standardized methodology. J Clin Microbiol 31: 406–409. 8381814 10.1128/jcm.31.2.406-409.1993 PMC262774

[26] Seki M, Honda I, Fujita I, Yano I, Yamamoto S, et al (2009) Whole genome sequence analysis of Mycobacterium bovis bacillus Calmette-Guerin (BCG) Tokyo 172: a comparative study of BCG vaccine substrains. Vaccine 27: 1710–1716. 19200449 10.1016/j.vaccine.2009.01.034

[27] Perez-Osorio AC, Boyle DS, Ingham ZK, Ostash A, Gautom RK, et al (2012) Rapid identification of mycobacteria and drug-resistant Mycobacterium tuberculosis by use of a single multiplex PCR and DNA sequencing. J Clin Microbiol 50: 326–336. 22162548 10.1128/JCM.05570-11 PMC3264146

[28] CLSI (2008) Laboratory detection and identification of mycobacteria: Approved guideline. CLSI document M48-A. Clinical and Laboratory Standards Institute, Wayne, PA.

[29] Eisenach KD, Cave MD, Bates JH, Crawford JT (1990) Polymerase chain reaction amplification of a repetitive DNA sequence specific for Mycobacterium tuberculosis. J Infect Dis 161: 977–981. 2109022 10.1093/infdis/161.5.977

[30] Thierry D, Brisson-Noel A, Vincent-Levy-Frebault V, Nguyen S, Guesdon JL, et al (1990) Characterization of a Mycobacterium tuberculosis insertion sequence, IS6110, and its application in diagnosis. J Clin Microbiol 28: 2668–2673. 2177747 10.1128/jcm.28.12.2668-2673.1990 PMC268253

[31] Flores LL, Pai M, Colford JM Jr, Riley LW (2005) In-house nucleic acid amplification tests for the detection of Mycobacterium tuberculosis in sputum specimens: meta-analysis and meta-regression. BMC Microbiol 5: 55. 16202138 10.1186/1471-2180-5-55 PMC1260021

[32] Savelkoul PH, Catsburg A, Mulder S, Oostendorp L, Schirm J, et al (2006) Detection of Mycobacterium tuberculosis complex with Real Time PCR: comparison of different primer-probe sets based on the IS6110 element. J Microbiol Methods 66: 177–180. 16427712 10.1016/j.mimet.2005.12.003

[33] Alonso H, Samper S, Martin C, Otal I (2013) Mapping IS6110 in high-copy number Mycobacterium tuberculosis strains shows specific insertion points in the Beijing genotype. BMC Genomics 14: 422. 23800083 10.1186/1471-2164-14-422 PMC3701491

[34] Huyen MN, Tiemersma EW, Kremer K, de Hass P, Lan NT, et al (2013) Characterisation of Mycobacterium tuberculosis isolates lacking IS6110 in Viet Nam. Int J Tuberc Lung Dis 17: 1479–1485. 24125454 10.5588/ijtld.13.0149

[35] Fomukong NG, Tang TH, al-Maamary S, Ibrahim WA, Ramayah S, et al (1994) Insertion sequence typing of Mycobacterium tuberculosis: characterization of a widespread subtype with a single copy of IS6110. Tuber Lung Dis 75: 435–440. 7718832 10.1016/0962-8479(94)90117-1

[36] Behr MA, Warren SA, Salamon H, Hopewell PC, Ponce de LA, et al (1999) Transmission of Mycobacterium tuberculosis from patients smear-negative for acid-fast bacilli. Lancet 353: 444–449. 9989714 10.1016/s0140-6736(98)03406-0

[37] van Soolingen D, Hermans PW, de Haas PE, van Embden JD (1992) Insertion element IS1081-associated restriction fragment length polymorphisms in Mycobacterium tuberculosis complex species: a reliable tool for recognizing Mycobacterium bovis BCG. J Clin Microbiol 30: 1772–1777. 1352785 10.1128/jcm.30.7.1772-1777.1992 PMC265379

[38] Collins DM, Stephens DM (1991) Identification of an insertion sequence, IS1081, in Mycobacterium bovis. FEMS Microbiol Lett 67: 11–15. 1663885 10.1016/0378-1097(91)90435-d

[39] Cole ST, Brosch R, Parkhill J, Garnier T, Churcher C, et al (1998) Deciphering the biology of Mycobacterium tuberculosis from the complete genome sequence. Nature 393: 537–544. 9634230 10.1038/31159

[40] Altschul SF, Gish W, Miller W, Myers EW, Lipman DJ (1990) Basic local alignment search tool. J Mol Biol 215: 403–410. 2231712 10.1016/S0022-2836(05)80360-2

[41] Van Deun A, Salim AH, Cooreman E, Daru P, Das AP, et al (2004) Scanty AFB smears: what's in a name? Int J Tuberc Lung Dis 8: 816–823. 15260271

[42] Miller DN, Bryant JE, Madsen EL, Ghiorse WC (1999) Evaluation and optimization of DNA extraction and purification procedures for soil and sediment samples. Appl Environ Microbiol 65: 4715–4724. 10543776 10.1128/aem.65.11.4715-4724.1999 PMC91634

[43] Drain PK, Hyle EP, Noubary F, Freedberg KA, Wilson D, et al (2013) Diagnostic point-of-care tests in resource-limited settings. Lancet Infect Dis 10.1016/S1473-3099(13)70250-0 PMC4016042 24332389

[44] Denkinger CM, Pai M (2011) Point-of-care tuberculosis diagnosis: are we there yet? Lancet Infect Dis 10.1016/S1473-3099(11)70257-2 22015304

