# Supplementary figures and images for: Rapid Detection of Mycobacterium tuberculosis by Recombinase Polymerase Amplification
Source: PLoS One. 2014 Aug 13;9(8):e103091. doi: 10.1371/journal.pone.0103091 (PMC4138011; doi:10.1371/journal.pone.0103091)

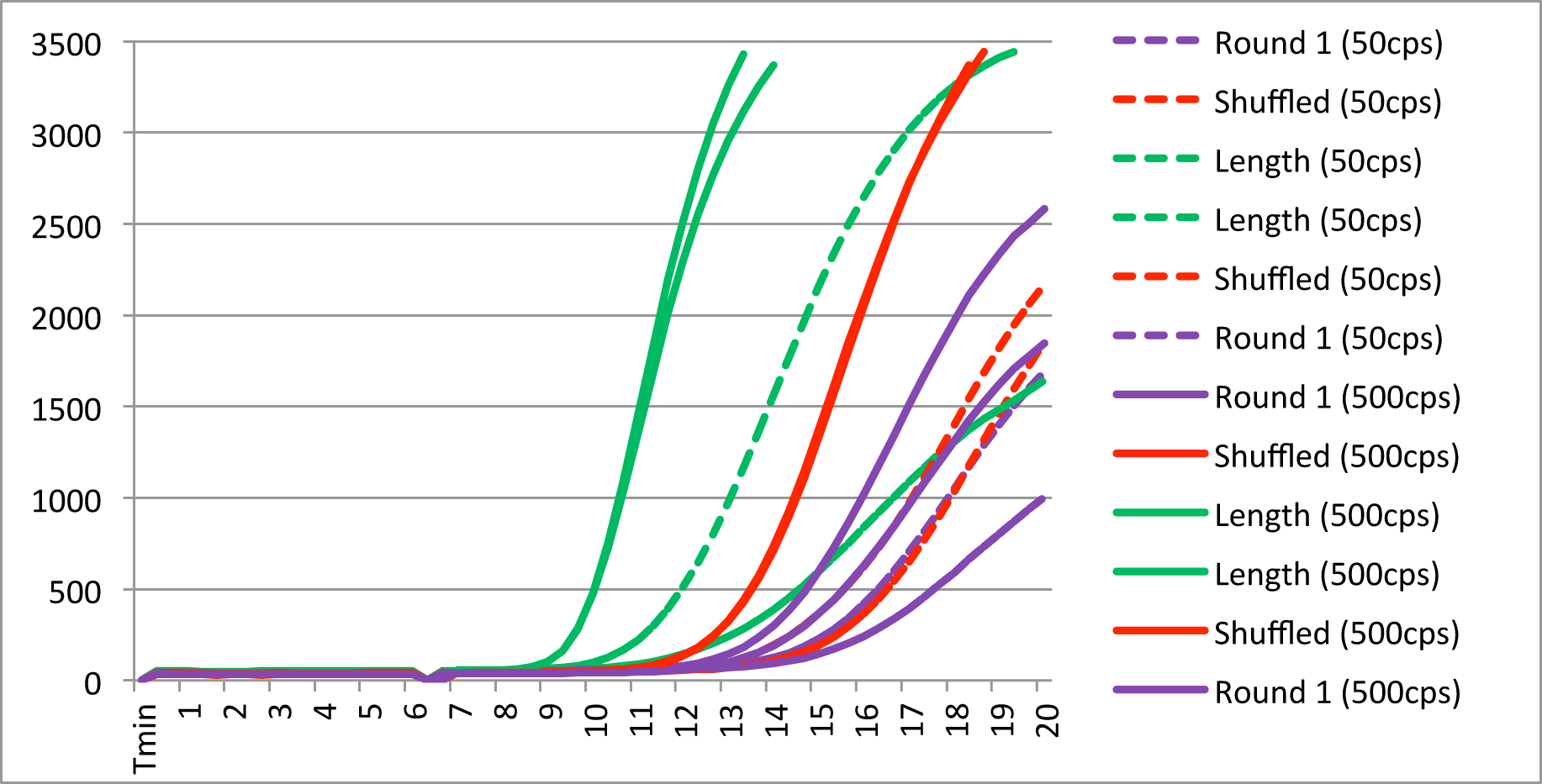

Supplement: Figure S1 — Benefits of RPA primer optimisation. This figure shows a side-by-side comparison of duplicates of the optimal primer pairs chosen during each round of screening for IS6110 with either 50 (dotted lines) or 500 (solid lines) copies of template. The primers chosen at the end of the first round of screening (purple, ‘Round 1’), can detect both 50 and 500 copies of template. The optimal primers identified by shuffling these primers upstream and downstream in single base increments (red, ‘Shuffled’) give faster detection times and result in higher levels of fluorescence. The primers chosen from lengthening and shortening the 3′ end of these shuffled primers (green, ‘Length’) show further improvement to detection time and fluorescent signals. Note that in all instances, poor duplicates are often indicative that an assay is close to its limit of detection. (TIF) [file pone.0103091.s001.tif]
