# Supplementary material for: Rapid Detection of Mycobacterium tuberculosis by Recombinase Polymerase Amplification
Source: PLoS One. 2014 Aug 13;9(8):e103091. doi: 10.1371/journal.pone.0103091 (PMC4138011; doi:10.1371/journal.pone.0103091)
Supplement: Table S1 — Culture, smear and RPA test results derived from clinical specimens. The scores for bacteria counted in the smear +ve specimens are shown in parenthesis, e.g. +ve (2+). Smear scores that were scanty are shown as +/−. Acronyms and other points. MTBC – Mycobacterium tuberculosis complex; NT – Not Tested; NTM = - Non Tuberculous Mycobacteria. MTBC. * Primary culture was contaminated; specimen was confirmed via a fresh specimen; # bronchoalveolar lavage (others not marked are sputum). (DOCX) [file pone.0103091.s002.docx]

| **Sample #** | **Culture** | **Smear** | ***IS*6110** | ***IS*1081** | **Sample #** | **Culture** | **Smear** | ***IS*6110** | ***IS*1081** | **Sample #** | **Culture** | **Smear** | ***IS*6110** | ***IS*1081** |
| --- | --- | --- | --- | --- | --- | --- | --- | --- | --- | --- | --- | --- | --- | --- |
| 1 | MTBC | +ve (1+) | +ve | +ve | 42 | MTBC | +ve (4+) | NT | +ve | 83 | NTM | -ve | NT | -ve |
| 2 | MTBC | +ve (1+) | +ve | +ve | 43 | MTBC | +ve (4+) | NT | +ve | 84 | NTM | -ve | NT | -ve |
| 3 | MTBC | +ve (1+) | +ve | +ve | 44 | MTBC | +ve (4+) | +ve | +ve | 85 | -ve | -ve | +ve | NT |
| 4 | MTBC | +ve (1+) | +ve | -ve | 45# | MTBC | +ve (4+) | +ve | NT | 86 | -ve | -ve | -ve | -ve |
| 5 | MTBC | +ve (1+) | +ve | NT | 46# | MTBC | +ve (4+) | +ve | NT | 87 | -ve | -ve | -ve | -ve |
| 6 | MTBC | +ve (1+) | +ve | NT | 47 | MTBC | +ve (4+) | +ve | NT | 88 | -ve | -ve | -ve | -ve |
| 7 | MTBC | +ve (1+) | +ve | NT | 48 | MTBC | +ve (4+) | +ve | NT | 89 | -ve | -ve | -ve | NT |
| 8 | MTBC | +ve (1+) | +ve | NT | 49 | MTBC | +ve (4+) | +ve | NT | 90 | -ve | -ve | -ve | NT |
| 9 | MTBC | +ve (1+) | NT | +ve | 50 | MTBC | +ve (4+) | +ve | NT | 91 | -ve | -ve | -ve | NT |
| 10 | MTBC | +ve (2+) | +ve | +ve | 51 | MTBC | +ve (4+) | +ve | NT | 92 | -ve | -ve | -ve | NT |
| 11 | MTBC | +ve (2+) | +ve | NT | 52 | MTBC | -ve | +ve | +ve | 93 | -ve | -ve | -ve | NT |
| 12 | MTBC | +ve (2+) | +ve | NT | 53 | MTBC | -ve | +ve | NT | 94 | -ve | -ve | -ve | NT |
| 13 | MTBC | +ve (2+) | +ve | NT | 54 | MTBC | -ve | +ve | NT | 95 | -ve | -ve | -ve | NT |
| 14 | MTBC | +ve (2+) | +ve | NT | 55 | MTBC | -ve | +ve | NT | 96 | -ve | -ve | -ve | NT |
| 15 | MTBC | +ve (2+) | +ve | NT | 56 | MTBC | -ve | +ve | NT | 97 | -ve | -ve | -ve | NT |
| 16 | MTBC | +ve (2+) | NT | +ve | 57 | MTBC | -ve | +ve | NT | 98 | -ve | -ve | -ve | NT |
| 17 | MTBC | +ve (2+) | NT | +ve | 58 | MTBC | -ve | +ve | NT | 99 | -ve | -ve | -ve | NT |
| 18 | MTBC | +ve (2+) | NT | +ve | 59 | MTBC | -ve | +ve | NT | 100 | -ve | -ve | -ve | NT |
| 19 | MTBC | +ve (2+) | NT | +ve | 60 | MTBC | -ve | -ve | +ve | 101 | -ve | -ve | -ve | NT |
| 20 | MTBC | +ve (2+) | NT | +ve | 61 | MTBC | -ve | -ve | -ve | 102 | -ve | -ve | NT | -ve |
| 21 | MTBC | +ve (2+) | NT | +ve | 62 | MTBC | -ve | -ve | NT | 103 | -ve | -ve | NT | -ve |
| 22 | MTBC | +ve (2+) | NT | +ve | 63 | MTBC | -ve | -ve | NT | 104 | -ve | -ve | NT | -ve |
| 23 | MTBC | +ve (2+) | NT | +ve | 64 | MTBC | -ve | -ve | NT | 105 | -ve | -ve | NT | -ve |
| 24 | MTBC | +ve (3+) | +ve | +ve | 65 | MTBC | -ve | -ve | NT | 106 | -ve | -ve | NT | -ve |
| 25 | MTBC | +ve (3+) | +ve | +ve | 66 | MTBC | -ve | NT | -ve | 107 | -ve | -ve | NT | -ve |
| 26 | MTBC | +ve (3+) | +ve | +ve | 67 | MTBC | -ve | NT | -ve | 108 | -ve | +ve (1+) | -ve | -ve |
| 27 | MTBC | +ve (3+) | +ve | +ve | 68 | MTBC* | +ve (1+) | +ve | NT | 109 | -ve | -ve | -ve | -ve |
| 28 | MTBC | +ve (3+) | +ve | +ve | 69 | MTBC* | +ve (3+) | +ve | NT | 110 | -ve | -ve | +ve | -ve |
| 29 | MTBC | +ve (3+) | +ve | NT | 70 | MTBC* | +ve (3+) | +ve | NT | 111 | -ve | -ve | -ve | -ve |
| 30 | MTBC | +ve (3+) | +ve | NT | 71 | NTM | +ve (+/-) | -ve | -ve | 112 | -ve | -ve | -ve | -ve |
| 31 | MTBC | +ve (3+) | +ve | NT | 72 | NTM | +ve (+/-) | -ve | -ve | 113 | -ve | -ve | -ve | -ve |
| 32 | MTBC | +ve (3+) | +ve | NT | 73 | NTM | +ve (+/-) | -ve | NT | 114 | -ve | -ve | -ve | -ve |
| 33 | MTBC | +ve (3+) | NT | +ve | 74 | NTM | +ve (1+) | -ve | -ve | 115 | -ve | -ve | -ve | -ve |
| 34 | MTBC | +ve (3+) | NT | +ve | 75 | NTM | -ve | -ve | -ve | 116 | -ve | -ve | -ve | -ve |
| 35 | MTBC | +ve (3+) | NT | +ve | 76 | NTM | -ve | -ve | -ve | 117 | -ve | -ve | -ve | -ve |
| 36 | MTBC | +ve (3+) | NT | +ve | 77 | NTM | -ve | -ve | -ve | 118 | -ve | -ve | -ve | -ve |
| 37 | MTBC | +ve (3+) | NT | +ve | 78 | NTM | -ve | -ve | -ve | 119 | -ve | -ve | -ve | -ve |
| 38 | MTBC | +ve (4+) | NT | +ve | 79 | NTM | -ve | -ve | -ve | 120 | -ve | -ve | -ve | -ve |
| 39 | MTBC | +ve (4+) | NT | +ve | 80 | NTM | -ve | -ve | -ve | 121 | -ve | -ve | -ve | -ve |
| 40 | MTBC | +ve (4+) | NT | +ve | 81 | NTM | -ve | -ve | NT |  |  |  |  |  |
| 41 | MTBC | +ve (4+) | NT | +ve | 82 | NTM | -ve | NT | -ve |  |  |  |  |  |
